# Stable reconstruction of the (110) surface and its role in pseudocapacitance of rutile-like RuO_2_

**DOI:** 10.1038/s41598-017-10331-z

**Published:** 2017-09-04

**Authors:** Hayk A. Zakaryan, Alexander G. Kvashnin, Artem R. Oganov

**Affiliations:** 1 Yerevan State University, 1 Alex Manoogian St., 0025 Yerevan, Armenia; 2 Skolkovo Institute of Science and Technology, Skolkovo Innovation Center, 143026, 3 Nobel Street, Moscow, Russian Federation; 3 Moscow Institute of Physics and Technology, 141700, 9 Institutsky lane, Dolgoprudny, Russian Federation; 4 Department of Geosciences and Center for Materials by Design, Institute for Advanced Computational Science, State University of New York, Stony Brook, NY 11794-2100 USA; 5 International Center for Materials Discovery, Northwestern Polytechnical University, Xi’an, 710072 China

## Abstract

Surfaces of rutile-like RuO_2_, especially the most stable (110) surface, are important for catalysis, sensing and charge storage applications. Structure, chemical composition, and properties of the surface depend on external conditions. Using the evolutionary prediction method USPEX, we found stable reconstructions of the (110) surface. Two stable reconstructions, RuO_4_–(2 × 1) and RuO_2_–(1 × 1), were found, and the surface phase diagram was determined. The new RuO_4_–(2 × 1) reconstruction is stable in a wide range of environmental conditions, its simulated STM image perfectly matches experimental data, it is more thermodynamically stable than previously proposed reconstructions, and explains well pseudocapacitance of RuO_2_ cathodes.

## Introduction

In the era of nanotechnology, steady miniaturization of electronic devices to nanometer scale takes place, with quantum and surface effects playing a major role for properties. Surface science becomes crucial for future. One of the most studied materials for catalysis, sensing and energy applications is the rutile-type RuO_2_^1^. Many researchers studied catalytic properties of RuO_2_ to enhance its catalytic efficiency for the oxidation of CO, NO and other molecules, which are important in industry^2–4^. In sensing devices, ruthenium is often used as a dopant for rutile-type SnO_2_. Ruthenium oxide is used in many applications as a thin layer to enhance sensitivity and selectivity of devices^5, 6^. It was also used as a cathode material for supercapacitors, displaying constant capacitance over the wide range of electric potentials^7^.

All these applications rely on unique properties of ruthenium dioxide. Under normal conditions RuO_2_ has tetragonal rutile-type structure, with *P4*_2_ */mnm* space group with two ruthenium and four oxygen atoms in the unit cell^8, 9^. Under high pressures RuO_2_ transforms to a CaCl_2_-type phase at 6 GPa^10^ and to pyrite structure at 82 GPa^11^.

There are also several known ruthenium oxides: RuO_4_, RuO and RuO_3_^12^. RuO exists in a gas phase at temperatures above 1900 K^12, 13^. Ruthenium trioxide (RuO_3_) exists in a gaseous form in the temperature range from 1300 to 2000 K, while the solid state of RuO_3_ forms only on substrates, i.e. on quartz surface at 400 K^12^. Ruthenium tetroxide can be in a gas, liquid or solid states. Below 1300 K the gaseous ruthenium tetroxide (RuO_4_) is formed^12^, which condenses at temperatures below 300 K ^12, 14^.

A number of theoretical and experimental works were devoted to detailed investigation of different surfaces of RuO_2_^15–17^. It was found that at ambient conditions the most stable RuO_2_ surface has (110) crystallographic orientation^18^. However, the atomic structure and even the composition of the surface can be changed under different environmental conditions (temperature and partial pressure of oxygen)^18^. Several theoretical predictions of possibly stable terminations of (110)-RuO_2_ surface were made by Reuter *et al*.^19, 20^.

Scanning tunneling microscopy (STM) is often used to study surfaces of materials^15^. However, it shows only the top layers of the materials and in a case of RuO_2_ only oxygen can be distinguished^15^. Thus, the actual structure of the surface becomes largely hidden from the eye of the experimentalist. Due to the fact that (110)-RuO_2_ surface is very sensitive to environmental conditions, the atomic structure and stoichiometry of the (110) surface may change drastically^15^.

RuO_2_ is the most widely used material in pseudocapacitors, novel energy storage devices which are in great demand for different applications. The pseudocapacitive behavior of RuO_2_ was first studied and explained by Trasatti and Buzzanca^21^. In their paper, it was proposed that the main mechanism of charge storage can be explained by the following redox reaction:

1 $$Ru{O}_{x}{(OH)}_{y}+\delta {H}^{+}+\delta {e}^{-}\leftrightarrow Ru{O}_{x-\delta }{(OH)}_{y+\delta }\cdot $$

Supercapacitive behavior occurs due to proton-electron double insertion. Thus each adsorbed or intercalated hydrogen atom (proton) will induce pseudocapacitance in the cathode material. Despite intense research devoted to the study of hydrogen intercalation in the cathode materials^22–29^, the atomic-scale processes are still not clearly understood. One of the main problems is the influence of proton adsorption, because it is difficult to distinguish surface pseudocapacitance (charge stored due to proton intercalation into the material) from double layer capacitance (charge stored due to electrostatic potential between electrode surface and electrolyte). The energetics of proton intercalation, atomic structure and stability of hydrogenated surface are still uncertain. For all of these problems, an investigation of possible surface reconstructions is essential.

It should be noted that none of prior theoretical predictions used global optimization techniques to find the most stable reconstructions of the (110)-RuO_2_ surface. Using evolutionary structure prediction algorithm USPEX^30–33^ and density functional theory we discovered new reconstructions of the (110)-RuO_2_ surface. This allows clearer explanations and deeper understanding of the processes occurring on surfaces. The formation conditions of studied reconstructions were estimated by the calculations of the surface energy as a function of oxygen chemical potential. Obtained phase diagram gives stability fields of different reconstructions in terms of various environmental conditions (oxygen partial pressure and temperature). Calculated voltage for adsorption of hydrogen on the new (110)-RuO_2_ surface reconstructions will answer the question “how the surface redox reaction contributes to pseudocapacitance of RuO_2_ electrode?”

## Results

**Figure 1:**
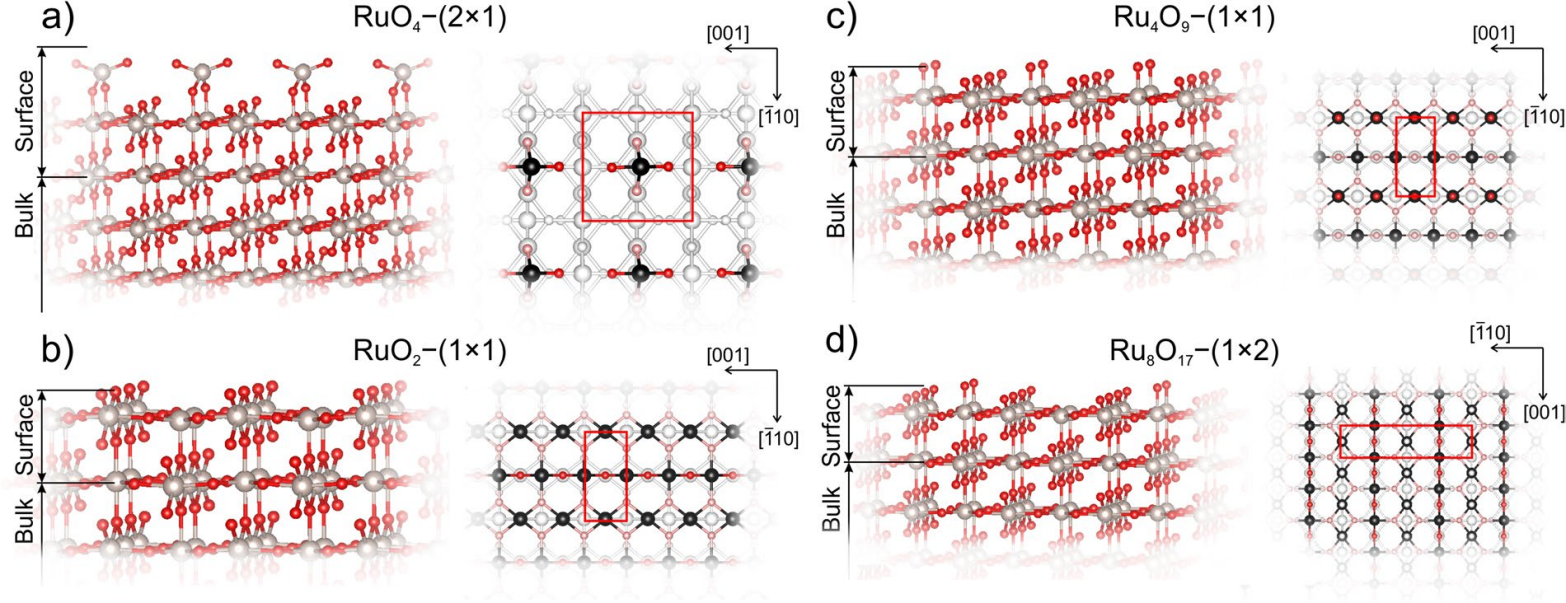
Predicted new reconstructions of (110)-RuO_2_ surface: stable (**a**) RuO_4_–(2 × 1), (**b**) RuO_2_–(1 × 1), and closest to convex hull metastable (**c**) Ru_4_O_9_–(1 × 1) and (**d**) Ru_8_O_17_–(1 × 2). In the top views the Ru atoms of the top layer are black, oxygen atoms of the upper layer are red, oxygen atoms following the top layer are light red.

We searched for stable reconstructions of (110)-RuO_2_ surface using variable-composition evolutionary algorithm USPEX adapted for surfaces^33^. We predicted several reconstructions, shown in Fig. 1. It is important to note that all these reconstructions have the same substrate, and the surface reconstruction takes place on top of the substrate, in the thickness region 3–5 Å.

**Figure 2:**
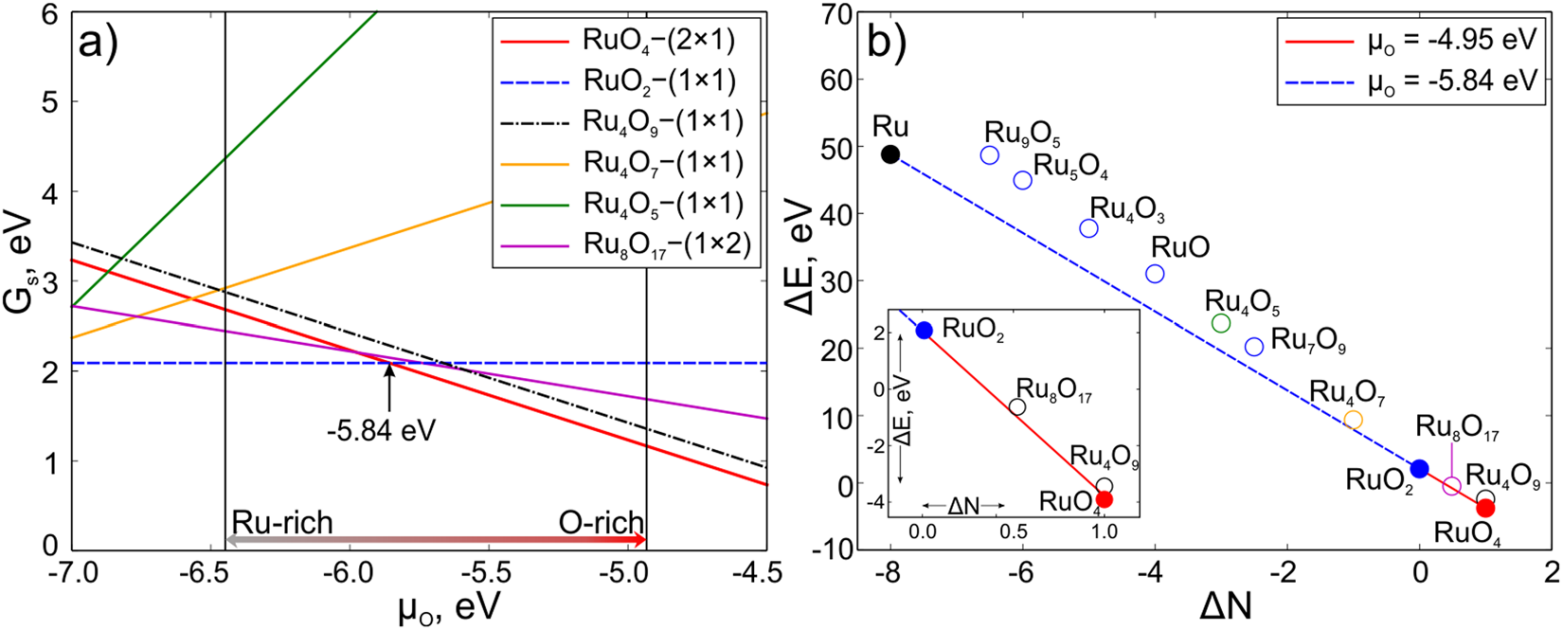
(**a**) Surface energy per unit cell as a function of oxygen chemical potential (µ_O_). (**b**) Convex hull of (110)-RuO_2_ reconstructions. Color of points corresponds to the color of lines in (a). The inset zooms in on the region of Δ*N* from −0.5 to 1.5.

We found 2 stable and 2 metastable reconstructions, which are closest to convex hull (see Fig. 2b). Different reconstructions of (110)-RuO_2_were denoted as RuO_4_–(2 × 1) (Fig. 1a), RuO_2_–(1 × 1) (Fig. 1b), Ru_4_O_9_–(1 × 1) (Fig. 1c) and Ru_8_O_17_–(1 × 2) (Fig. 1d). The nomenclature of the predicted reconstructions reflects the stoichiometry of reconstructed surface regions. The stoichiometry of the surface region equals the difference between stoichiometry of the entire system minus stoichiometry of the substrate. Number in the brackets is the number of surface cells in the reconstructed cell. The total number of the atoms in considered structures can be found in Table 1.

**Table 1:** Predicted surface reconstructions. Number of ruthenium and oxygen atoms (*N*_Ru_, *N*_O_), number of multiplications of the unit cell (*N*_cell_), total energy per cell from DFT calculations (*E*_total_), Δ*N* and Δ*E* values.

Structure	*N*_Ru_	*N*_O_	*N*_cell_	*E*_total_, eV	Δ*N*	Δ*E*, eV
RuO_4_–(2 × 1)	17	36	2	−383.24	1	−3.77
Ru_4_O_9_–(1 × 1) “cusp”	8	17	1	−180.39	1	−3.59
Ru_8_O_17_–(1 × 2)	16	33	2	−355.17	0.5	−0.78
RuO_2_–(1 × 1)	8	16	1	−174.73	0	2.07

Three of predicted reconstructions have already been known from previous theoretical studies: RuO_2_–(1 × 1), Ru_4_O_9_–(1 × 1) and Ru_8_O_17_–(1 × 2)^20, 34^. Reconstruction RuO_4_–(2 × 1) is newly predicted. It is interesting to note that among all predicted reconstructions we found one (Ru_4_O_5_–(1 × 1)), which contains a RuO monolayer on top of the RuO_2_ substrate.

Let us now move to investigation of stability of predicted surface reconstructions. Using equation (5) we calculated the surface energy of all predicted surface reconstructions as a function of oxygen chemical potential, shown in Fig. 2a. The structure with the lowest surface energy in a given range of chemical potentials is deemed stable at those chemical potentials. The range of oxygen chemical potential from −6.45 to −4.95 eV is experimentally achievable (see Eq. (6)). For the convenience of readers, we placed all the equations in the Methods section in the end of the paper. Chemical potential lower than −6.45 eV will lead to desorption of oxygen and pure ruthenium will precipitate. Values µ_O_ > −4.95 eV indicate the formation of oxygen molecules (O_2_) on the surface. As one can see from Fig. 2a, there are two stable surface reconstructions: RuO_2_–(1 × 1) and RuO_4_–(2 × 1) (dashed blue and bold red lines in Fig. 2a). RuO_2_–(1 × 1) is stable in the range of oxygen chemical potentials from −6.45 to −5.84 eV and has bulk stoichiometry and bulk-like termination (Ru:O = 1:2). The other stable structure is RuO_4_–(2 × 1), which has the lowest surface energy (*G*_s_) in the range of µ_O_ from −5.84 to −4.95 eV (red line in Fig. 2a). This reconstruction has one four-coordinate Ru atom and 4 oxygen atoms, two of which are two-coordinate and the other two are one-coordinate (Fig. 1a). According to ref. 20, another reconstruction called “Cusp” (in our study it is Ru_4_O_9_–(1 × 1) due to another nomenclature) should be stable in the same range as our RuO_4_–(2 × 1). Ru_4_O_9_–(1 × 1) has bulk-like termination with one additional oxygen atom located on top of 5-coordinate Ru atom (see red atom in the top view of Fig. 1c). We found that Ru_4_O_9_–(1 × 1) reconstruction has surface energy higher than RuO_4_–(2 × 1) by 0.1 eV (see dotted line in Fig. 2a) and therefore is metastable. Here and below all energy values are taken per unit cell. It is important that RuO_4_–(2 × 1) and Ru_4_O_9_–(1 × 1) reconstructions have the same Δ*N*, which leads to the same slopes of *G*_s_(µ_O_) functions (they are parallel). Values of Δ*N* and Δ*E* for all predicted surface reconstructions calculated by using eq. (7) are presented in Table 1. Additional calculations with a doubled substrate thickness along *c*-axis gave the same result, i.e. stability of RuO_4_–(2 × 1) versus Ru_4_O_9_–(1 × 1).

The convex hull diagram for all considered structures is shown in Fig. 2b, where each point represents one structure. Solid points represent thermodynamically stable reconstructions, which form the convex hull. Metastable reconstructions are open circles and are located above the convex hull. Here only two reconstructions are found to be stable, namely RuO_2_–(1 × 1) and RuO_4_–(2 × 1). Ru_8_O_17_–(1 × 1) and Ru_4_O_9_–(1 × 1) are located very close to the convex hull line just by 0.06 and 0.1 eV above it, respectively.

The metastable reconstruction Ru_8_O_17_–(1 × 2) is geometrically similar to Ru_4_O_9_–(1 × 1), but with doubled cell in the $[\bar{1}10]$ direction, and one oxygen removed from a site above Ru (see Fig. 1d). This reconstruction has energy 0.06 eV above the convex hull (see Fig. 2b). Ru_4_O_7_–(1 × 1) is unstable, as was already shown in previous studies^20^.

**Figure 3:**
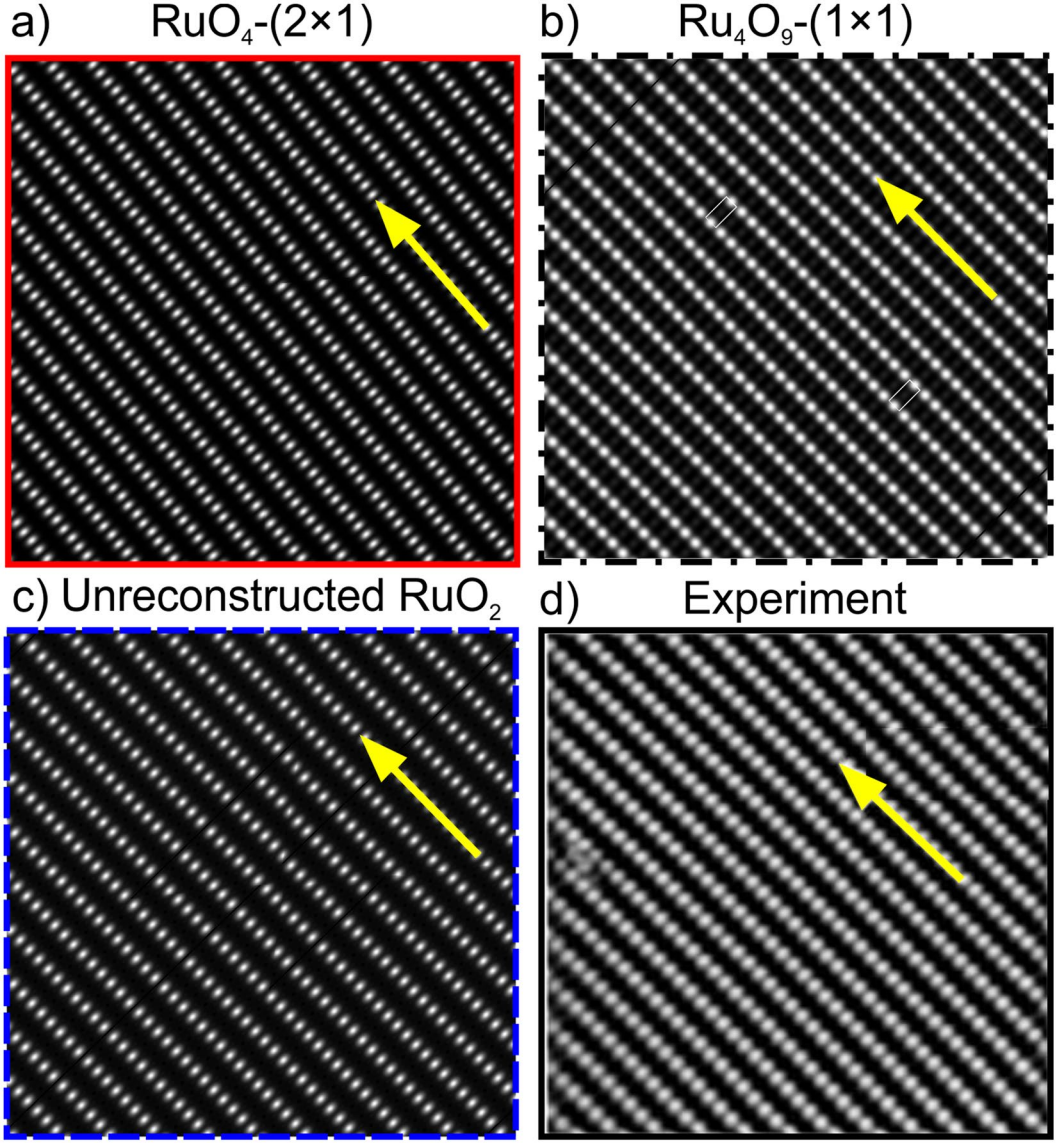
(**a**) Simulated STM images of RuO_4_–(2 × 1) and (**b**) Ru_4_O_9_–(1 × 1) reconstructions; (**c**) STM image of stoichiometric RuO_2_ (110) surface; (**d**) experimental STM image of RuO_2_ surface from ref. 36. The [001] direction is highlighted by yellow arrows.

To discriminate between structural models, we use the results of Scanning Transmission Microscopy (STM). We simulated the STM images of RuO_4_–(2 × 1), RuO_2_–(1 × 1) and Ru_4_O_9_–(1 × 1) reconstructions as the most stable ones. The comparison between them and experimental STM image of RuO_2_(110) surface was made. In Fig. 3a, simulated STM image of RuO_4_–(2 × 1) is presented, where bright dots are one-coordinate oxygen atoms. The distance between the atoms along the [001] direction (yellow arrow in Fig. 3a) is 3.2 Å, while the distance in the perpendicular direction (between the rows of atoms) is 6.26 Å. STM image of Ru_4_O_9_–(1 × 1) reconstruction is shown in Fig. 3b, where the distance along the [001] direction is 3.2 Å, in the perpendicular direction the distance equals to 6.4 Å. The simulated STM image of RuO_2_–(1 × 1) is in Fig. 3c, where distances along [001] direction and perpendicular to it are 3.2 and 6.4 Å, respectively. These images agree well with experimental data, where the corresponding distances are 3.12 and 6.38 Å, respectively^35, 36^ (see Fig. 3d). One must admit that all three models generate STM images consistent with experiment, which makes them difficult to distinguish from each other in experiments. While equally consistent with experimental STM images, our RuO_4_–(2 × 1) reconstruction is lower in energy and therefore is preferable.

**Figure 4:**
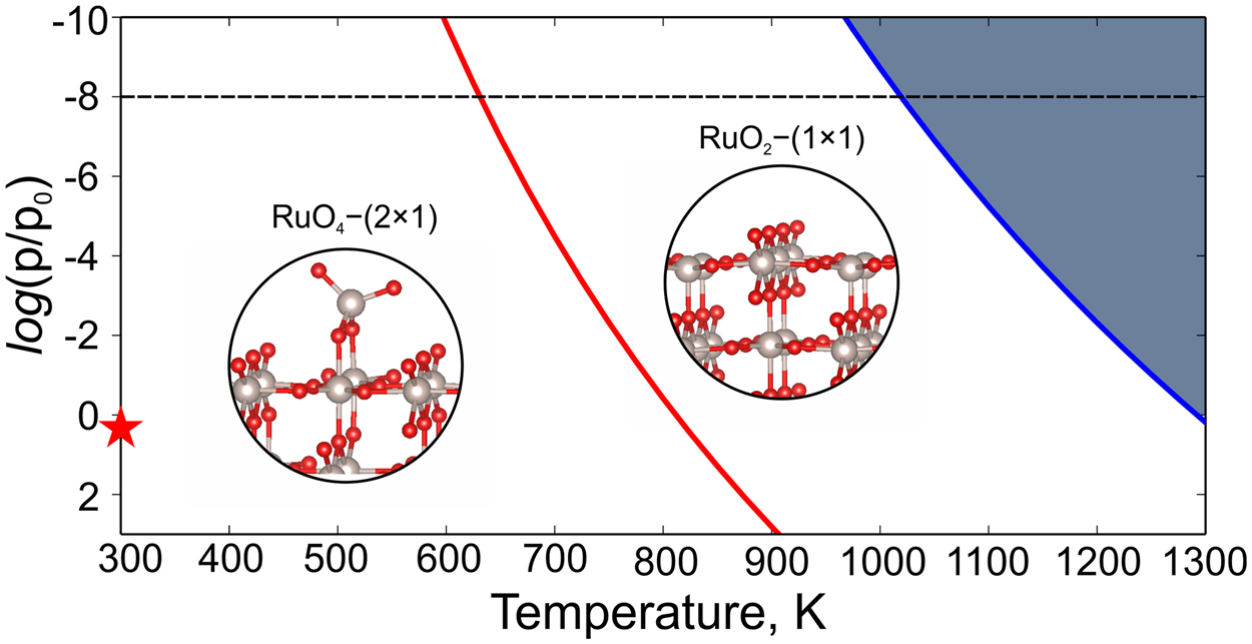
Surface phase diagram of (110)-RuO_2_. The dark region corresponds to the deposition of Ru metal. The *x*-axis is temperature; *y*-axis is oxygen partial pressure. The star denotes ambient conditions (*p* ≈ 0.21 atm, *T* = 273 K).

To determine stability fields of each surface reconstruction, we calculated the pressure-temperature phase diagram, shown in Fig. 4. Such phase diagram shows environmental conditions (partial pressure and temperature), suitable for the formation of new reconstructions. Both partial pressure of oxygen and temperature both enter the expression for the chemical potential:

2 $${\mu }_{O}=\frac{1}{2}\,[{E}_{{O}_{2}}+{{\rm{\Delta }}H}_{{O}_{2}}(T,{P}_{0})-{TS}_{{O}_{2}}(T,{P}_{0})+{k}_{B}Tln(\frac{P}{{P}_{0}})]=\frac{1}{2}{E}_{{O}_{2}}+{\rm{\Delta }}{\mu }_{O}(T,P)\cdot $$

where ${E}_{{O}_{2}}$ is the static energy of the O_2_ molecule (computed from first principles), ${{\rm{\Delta }}H}_{{O}_{2}}(T,{P}_{0}),{TS}_{{O}_{2}}(T,{P}_{0})$ are thermal parts of the Gibbs free energy of the gas of oxygen molecules as a function of temperature and pressure, and it was taken from thermodynamic database^37^.

One can see from the calculated phase diagram (Fig. 4) that RuO_4_–(2 × 1) reconstruction is stable at higher values of oxygen partial pressure and lower temperatures than RuO_2_–(1 × 1). The phase boundary (red line in Fig. 2) was plotted using equation (2) with the value of chemical potential of oxygen equal to −5.84 eV. Increasing temperature to 800 K at the pressure of 10^−8^ bar (dashed horizontal line) will lead to the formation of RuO_2_–(1 × 1) reconstruction (see Fig. 4).This fact perfectly agrees with experimental results, where RuO_2_–(1 × 1) reconstruction forms at $log(p/{p}_{0})=-8$ and $T\ge 600\,K$ ^35, 38^. Further increase of temperature up to 1050 K leads to desorption of oxygen (see blue line in Fig. 4). Blue line was plotted using ${\mu }_{{\rm{O}}}=-6.45$ eV in equation (2). Oxygen chemical potential equal to $-6.45$ eV delineates the region where deposition of pure ruthenium is favored. Note that at ambient conditions our RuO_4_–(2 × 1) reconstruction is the one which is stable.

Thermal stability of the newly predicted RuO_4_–(2 × 1) reconstruction was studied by means of molecular dynamics (MD) simulations. MD simulations were carried out at temperatures of 500 and 1200 K using the Nosé–Hoover thermostat^39, 40^ with a time step of 1 fs for a total simulation period of 5 ps. During the simulations the atomic structure of RuO_4_–(2 × 1) surface reconstruction remains intact: essentially, only dynamical bendings of the O-Ru-O angles at the upper layer were observed. The RuO_4_–(2 × 1) surface reconstruction is thermally stable.

Let us now consider the pseudocapacitive properties of studied RuO_2_ reconstructions. Previous studies^28, 29^ concluded that surface redox reaction will not contribute to capacitance of cathode material, because the calculated voltage is above the oxygen evolution potential (OEP) for different numbers of adsorbed hydrogen atoms^28, 29^. However, experimental work^23^ reported that redox reaction should be responsible for pseudocapacitance. To resolve this, we calculated the voltages for Ru_4_O_9_–(1 × 1) and RuO_4_–(2 × 1) reconstructions and prove that surface redox reaction takes place on the new RuO_4_–(2 × 1) reconstruction. Here we considered OEP as a boundary value of voltage applied to the whole system. For ideal systems, where overpotentials are not considered, OEP is 1.23 V. If voltage, calculated by eq. (8), is less than 1.23 V, then one observes a predominant influence of hydrogen intercalation into the cathode surface, which would contribute to pseudocapacitance. If, on the other hand, the calculated voltage is >1.23 V, then water splitting takes place and no proton adsorption or intercalation happen.

**Figure 5:**
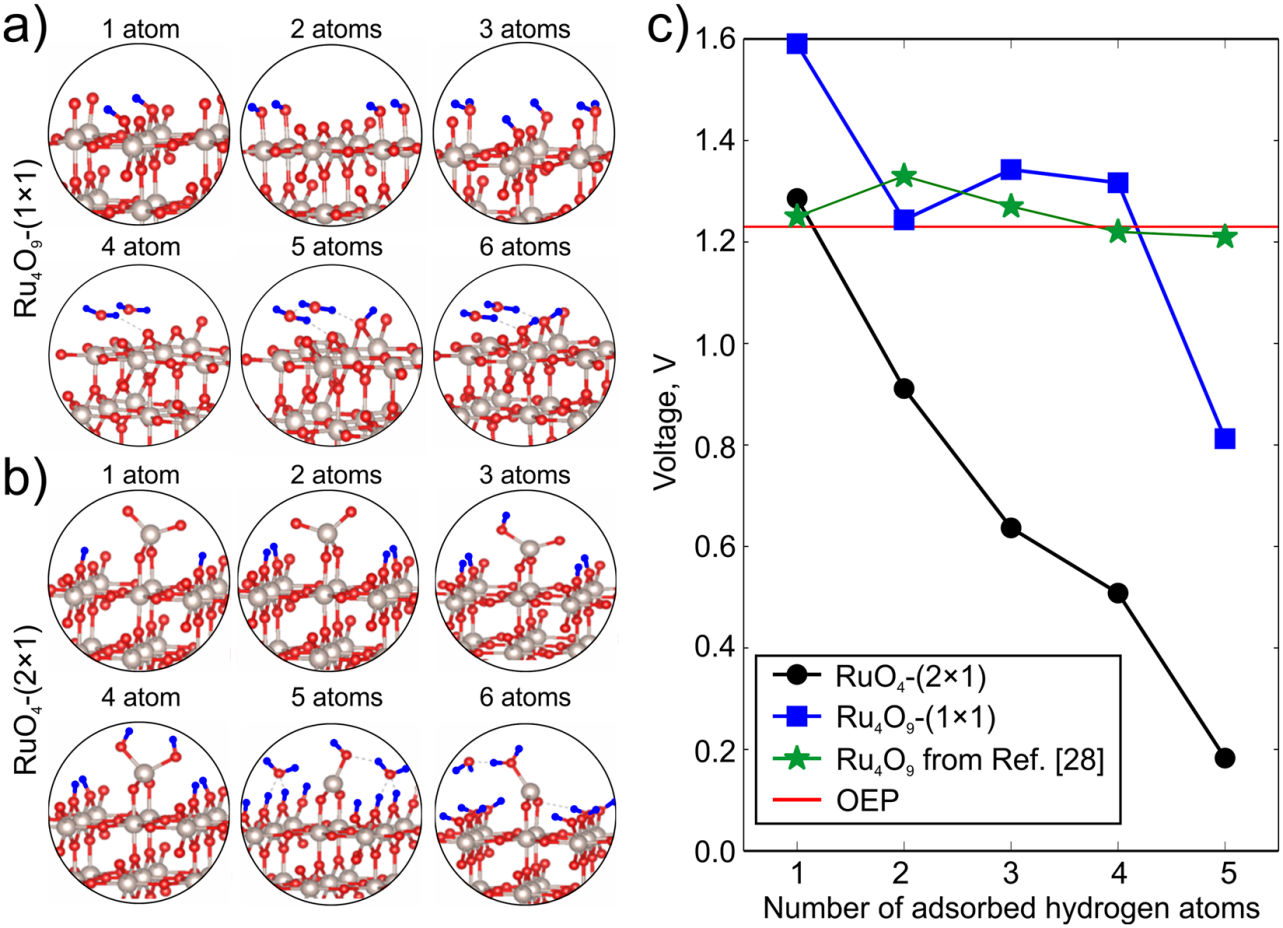
Configurations of adsorption sites of hydrogen atoms on the (**a**) Ru_4_O_9_–(1 × 1) and (**b**) RuO_4_–(2 × 1) reconstructions with the total number of hydrogens atoms from 1 to 6. Ru atoms are grey, oxygen is red, and hydrogen is blue; (**c**) Calculated voltage as a function of number of adsorbed hydrogen atoms for RuO_4_–(2 × 1) (black color) and Ru_4_O_9_–(1 × 1) (blue color). Green stars are reference data from ref. 29. Oxygen evolution potential is shown by horizontal red line.

To calculate the voltage, we considered adsorption of hydrogen atoms on the Ru_4_O_9_–(1 × 1) and RuO_4_–(2 × 1) surfaces. For the Ru_4_O_9_–(1 × 1) reconstruction, the most favorable positions of hydrogen atoms shown in Fig. 5a were taken from refs 28, 29 and 41. The energies of hydrogen adsorption agree well with ref. 41. All possible positions of hydrogen atoms (with the total number of atoms from 1 to 6) on the RuO_4_–(2 × 1) surface were considered, and only the most favorable ones are shown in Fig. 5b.

Using eqs (8) and (9) we calculated the voltages of RuO_4_–(2 × 1) reconstruction and Ru_4_O_9_–(1 × 1), compared to reference data from ref. 29 (see Fig. 5c). Calculated values of voltage for Ru_4_O_9_–(1 × 1) reconstruction are above or very close to OEP, which is in good agreement with ref. 29 (green stars in the Fig. 5c).

In stark contrast, RuO_4_–(2 × 1) reconstruction with more than one adsorbed hydrogen atoms shows voltage below the OEP. This means that our new RuO_4_–(2 × 1) reconstruction will adsorb hydrogen better than previously proposed^28^ Ru_4_O_9_–(1 × 1) (see blue curve with squares).Such behavior explains and confirms the contribution of the surface redox reaction to the pseudocapacitance of RuO_2_electrodes. We recall that RuO_4_–(2 × 1) reconstruction is the dominant one at normal conditions (Fig. 4).

In conclusion, we studied stable reconstructions of (110) surface of rutile RuO_2_using global optimization algorithm USPEX. We found several new reconstructions, as well as all previously proposed ones. Predicted stable RuO_4_–(2 × 1) reconstruction is found to be thermodynamically stable at normal conditions, and generally at oxygen-rich conditions. Simulated STM image of RuO_4_–(2 × 1) reconstruction perfectly matches the experimental STM image. Calculated voltage for adsorption of hydrogen on the new RuO_4_–(2 × 1) surface reconstruction is lower than oxygen evolution potential (OEP), and this result indicates the importance of the surface redox reaction to pseudocapacitance of RuO_2_ cathodes.

## Methods

Stable reconstructions of (110)-RuO_2_ surface were predicted using first-principles evolutionary algorithm (EA) as implemented in the USPEX code^30–33^, where 4 different multiplications of unit cell were considered, namely (1 × 1), (1 × 2), (2 × 1) and (2 × 2). Here, evolutionary searches were combined with structure relaxations using density functional theory (DFT)^42, 43^ within the spin-polarized generalized gradient approximation (Perdew-Burke-Ernzerhof functional)^44^, and the projector augmented wave method^45, 46^ as implemented in the VASP^47–49^ package. The plane–wave energy cutoff of 500 eV, and *k*-mesh of $0.05\times 2\pi /\AA$ resolution ensure excellent convergence of total energies. During structure search, the first generation was produced randomly, and succeeding generations were obtained by applying 40% heredity, 10% softmutation, 20% transmutation operations, respectively and 30% using random symmetric algorithm^50, 51^. Each of the considered supercells contained a vacuum layer of 15 Å and a substrate slab of 2 RuO_2_ layers (6 Å) with atoms in the topmost 3 Å allowed to relax. We also performed additional calculations of slabs with thickness increased up to 12 Å, and only the bottom layer was kept fixed to obtain more accurate surface energies for stable (110)-RuO_2_ reconstructions. No significant differences were found, which ensures reliability of our calculations.

For calculation of hydrogen adsorption on the predicted surface reconstructions, structure relaxation was carried out until the maximum net force on atoms became less than 0.01 eV/Å. The Monkhorst–Pack scheme^52^ was used to sample the Brillouin zone, using 6 × 6 × 1 *k*-points mesh and the plane–wave energy cutoff was set to 500 eV.

For variable-composition search of optimal surface reconstructions, it is important to set boundary values of chemical potentials, which are related to the free energies of bulk Ru, O_2_ molecule and bulk rutile-typeRuO_2_^53, 54^.

For the case of RuO_2_, the surface energy can be written in the following manner:

3 $${G}_{s}(T,P)=\frac{1}{N}[{G}^{slab}(T,P,{N}_{Ru},{N}_{O})-{N}_{Ru}{\mu }_{Ru}(T,P)-{N}_{O}{\mu }_{O}(T,P)],$$

where ${G}_{s}(T,P)$ is surface energy per unit cell, ${G}^{slab}(T,P,{N}_{Ru},{N}_{O})$ is the Gibbs free energy per cell of surface, which can be approximated as the total energy at 0 K^20^, *N* = *m* × *n* for an *m* × *n* surface supercell and serves as a normalization factor,$\,{N}_{Ru}$, ${\mu }_{Ru}$ and ${N}_{O}$, ${\mu }_{O}$ are the number and chemical potential of Ru and O atoms in the cell, respectively. In this approximation, temperature dependence is explicitly taken into account only for the chemical potential of oxygen (other values being much less dependent on temperature).

Chemical potentials in equilibrium with of RuO_2_ substrate are related through:

4 $${\mu }_{Ru}(T,P)+2{\mu }_{O}(T,P)={G}_{Ru{O}_{2}}(T,P),$$

where ${G}_{Ru{O}_{2}}(T,P)$ is Gibbs free energy of bulk RuO_2_. The surface energy can be recast in a form with only one variable chemical potential:

5 $${G}_{s}(T,P)=\frac{1}{N}\,[{G}^{slab}(T,P,{N}_{Ru},{N}_{O})-{N}_{Ru}{G}_{Ru{O}_{2}}^{bulk}(T,P)-({N}_{O}-2{N}_{Ru}){\mu }_{O}(T,P)]\cdot $$

Regarding physical bounds on chemical potentials, the chemical potential of Ru during crystallization on substrate was taken as lower limit and chemical potential when O_2_ molecule goes away from the substrate was taken as upper limit. So the final relation, which defines the physically meaningful range of chemical potentials, has the following form:

6 $$\frac{1}{2}{\rm{\Delta }}{G}_{f}(T,P)+\frac{1}{2}{E}_{{O}_{2}}\le {\mu }_{O}(T,P)\le \frac{1}{2}{E}_{{O}_{2}},$$

where ${E}_{{O}_{2}}$ is total energy of oxygen molecule, ${\rm{\Delta }}{G}_{f}(T,P)$ is the formation energy of bulk rutile-type RuO_2_ from gas phase, equals 3.3 eV, which is in a good agreement with experimental value of 3.16 eV at 1000 K^12, 55^. Above 1000 K the formation energy can reach the value of 3.2 eV.

Stability of different structures can be compared using equation (5) by plotting ${G}_{s}$ as a function of ${\mu }_{O}$ as shown in Fig. 2a. Each structure corresponds to a line on the phase diagram. A complementary and equivalent way to determine stability is to plot the convex hull diagram (see Fig. 2b), in Δ*E*-Δ*N* axes^33^, where

7 $${\rm{\Delta }}E=\frac{1}{N}\,[{G}^{slab}(T,P,{N}_{Ru},{N}_{O})-{N}_{Ru}{G}_{Ru{O}_{2}}^{bulk}(T,P)]\,{\rm{and}}\,{\rm{\Delta }}N=\frac{1}{N}({N}_{O}-2{N}_{Ru})\cdot $$

The calculation of electrode voltages was done using free energies of the surface with hydrogen adatoms on it^29, 41^. The voltage can be calculated using other methods, i.e. joint density functional theory (JDFT)^56^, which considers electrode-electrolyte interaction and overpotential influence. Another method considers *pH* and work function of surfaces^24^. However, all these methods strongly depend on the surface reconstruction. We calculate voltage of electrode, using method proposed by Liu *et al*.^29^, which can determine the contribution of redox reaction to pseudocapacitance. The voltage was calculated by using the following equation:

8 $$V(n)=-\frac{{\rm{\Delta }}{G}_{H}(n+1)-{\rm{\Delta }}{G}_{H}(n)}{{q}_{e}},$$

9 $${\rm{\Delta }}{G}_{H}(n)=\,{G}_{Ru{O}_{2}+nH}^{surf}-{G}_{Ru{O}_{2}}^{surf}-\frac{n}{2}{G}_{{H}_{2}},$$

where *n* is the number of adsorbed or intercalated hydrogen atoms, ${G}_{Ru{O}_{2}+nH}^{surf}$ is the free energy of surface with *n* adsorbed hydrogen atoms, ${G}_{Ru{O}_{2}}^{surf}$ is the surface free energy, ${G}_{{H}_{2}}\,$is free the energy of H_2_ molecule in a gas phase and *V*(*n*) is voltage as a function of the number of protons (hydrogen atoms) adsorbed on the surface or intercalated in the material. The voltage was calculated for the RuO_4_–(2 × 1) and doubled cell of Ru_4_O_9_–(1 × 1) due to different sizes of considered unit cells. The hydrogen atoms (from 1 to 6 atoms) were adsorbed on different positions as was done in previous studies^29, 41^. The calculated adsorption energies and voltages agree well with reference data^29, 41^.

## References

[1] Over H (2012). Surface chemistry of ruthenium dioxide in heterogeneous catalysis and electrocatalysis: from fundamental to applied research. Chem. Rev..

[2] Weaver JF (2013). Surface chemistry of late transition metal oxides. Chem. Rev..

[3] Wang H, Schneider WF (2007). Effects of coverage on the structures, energetics, and electronics of oxygen adsorption on RuO_2_ (110). J. Chem. Phys..

[4] Norskov JK, Abild-Pedersen F, Studt F, Bligaard T (2011). Density functional theory in surface chemistry and catalysis. Proc. Natl. Acad. Sci..

[5] Zhu Z (2009). Enhanced gas-sensing behaviour of Ru-doped SnO_2_ surface: A periodic density functional approach. J. Phys. Chem. Solids.

[6] Aroutiounian VM (2013). Study of the surface-ruthenated SnO_2_/MWCNTs nanocomposite thick-film gas sensors. Sens. Actuators B Chem..

[7] Conway BE, Pell WG (1997). & Tongchang Liu. Self-discharge and potential recovery phenomena at thermally and electrochemically prepared RuO_2_ supercapacitor electrodes. Electrochem. Acta.

[8] Mattheiss LF (1976). Electronic structure of RuO_2_, OsO_2_, and IrO_2_. Phys. Rev. B.

[9] Ze-Jin Y (2010). Electronic structure and optical properties of rutile RuO_2_ from first principles. Chin. Phys. B.

[10] Haines J, Léger JM (1993). Phase transitions in ruthenium dioxide up to 40 GPa: Mechanism for the rutile-tofluorite phase transformation and a model for the high-pressure behavior of stishovite SiO2. Phys. Rev. B.

[11] Tse JS (2000). Elastic properties of potential superhard phases of RuO_2_. Phys. Rev. B.

[12] Eichler B, Zude F, Fan W, Trautmann N, Herrmann G (1992). Volatilization and deposition of ruthenium oxides in a temperature gradient tube. Radiochim. Acta.

[13] Norman, J. H., Staley, H. G. & Bell, W. E. Mass spectrometric study of the noble metal oxides: ruthenium-oxygen system. in *Mass spectrometry in inorganic chemistry* (ed. Margrave, J. L.) **72**, 101–114 (AMERICAN CHEMICAL SOCIETY, 1968).

[14] Pley M, Wickleder MS (2005). Two crystalline modifications of RuO_4_. J. Solid State Chem..

[15] Rössler M, Günther S, Wintterlin J (2007). Scanning tunneling microscopy of the RuO_2_ (110) surface at ambient oxygen pressure. J. Phys. Chem. C.

[16] Over H (2000). Atomic-scale structure and catalytic reactivity of the RuO_2_ (110) surface.. Science.

[17] Pollak FH, Atanasoska L, Park HL (1984). Single crystal RuO_2_ (110): surface structure. J Electroanal Chem.

[18] Xu C (2014). Prediction on the surface phase diagram and growth morphology of nanocrystal ruthenium dioxide. J. Am. Ceram. Soc..

[19] Reuter K, Scheffler M (2003). Composition and structure of the RuO_2_ (110) surface in an O_2_ and CO environment: Implications for the catalytic formation of CO_2_. Phys. Rev. B.

[20] Reuter K, Scheffler M (2001). Composition, structure, and stability of RuO_2_ (110) as a function of oxygen pressure. Phys. Rev. B.

[21] Trasatti S (1991). Physical electrochemistry of ceramic oxides. Electrochimica Acta.

[22] Lin K-M, Chang K-H, Hu C-C, Li Y-Y (2009). Mesoporous RuO2 for the next generation supercapacitors with an ultrahigh power density. Electrochimica Acta.

[23] Sugimoto W, Yokoshima K, Murakami Y, Takasu Y (2006). Charge storage mechanism of nanostructured anhydrous and hydrous ruthenium-based oxides. Electrochimica Acta.

[24] Watanabe E, Rossmeisl J, Björketun ME, Ushiyama H, Yamashita K (2016). Atomic-scale analysis of the RuO_2_ water interface under electrochemical conditions. J. Phys. Chem. C.

[25] Fang Y-H, Liu Z-P (2010). Mechanism and tafel lines of electro-oxidation of water to oxygen on RuO_2_ (110). J. Am. Chem. Soc..

[26] Wei Y, Martinez U, Lammich L, Besenbacher F, Wendt S (2014). Atomic-scale view on the H_2_O formation reaction from H_2_ on O-rich RuO_2_ (110). J. Phys. Chem. C.

[27] Karlsson RKB, Cornell A, Pettersson LGM (2016). Structural changes in RuO_2_ during electrochemical hydrogen evolution. J. Phys. Chem. C.

[28] Ozoliņš V, Zhou F, Asta M (2013). Ruthenia-based electrochemical supercapacitors: insights from first-principles calculations. Acc. Chem. Res..

[29] Liu Y, Zhou F, Ozolins V (2012). Ab initio study of the charge-storage mechanisms in RuO_2_-based electrochemical ultracapacitors. J. Phys. Chem. C.

[30] Glass CW, Oganov AR, Hansen N (2006). USPEX—Evolutionary crystal structure prediction. Comput. Phys. Comm..

[31] Oganov AR, Glass CW (2006). Crystal structure prediction using ab initio evolutionary techniques: principles and applications. J. Chem. Phys..

[32] Oganov AR, Ma Y, Lyakhov AO, Valle M, Gatti C (2010). Evolutionary crystal structure prediction as a method for the discovery of minerals and materials. Rev. Mineral. Geochem..

[33] Zhu, Q., Li, L., Oganov, A. R. & Allen, P. B. Evolutionary method for predicting surface reconstructions with variable stoichiometry. *Phys. Rev. B* **87**, (2013).

[34] Wang H, Schneider WF, Schmidt D (2009). Intermediates and spectators in O_2_ dissociation at the RuO_2_ (110) Surface. J. Phys. Chem. C.

[35] Kim SH, Wintterlin J (2004). Atomic scale investigation of the oxidation of CO on RuO_2_ (110) by scanning tunneling microscopy. J. Phys. Chem. B.

[36] Over H (2004). Visualization of atomic processes on ruthenium dioxide using scanning tunneling microscopy. Chem. Phys. Chem..

[37] NIST, Chemistry WebBook. Available at: http://webbook.nist.gov/.

[38] He YB, Knapp M, Lundgren E, Over H (2005). Ru(0001) model catalyst under oxidizing and reducing reaction conditions: *In-situ* high-pressure surface X-ray diffraction study. J. Phys. Chem. B.

[39] Nosé S (1984). A unified formulation of the constant temperature molecular dynamics methods. J. Chem. Phys..

[40] Hoover WG (1985). Canonical dynamics: Equilibrium phase-space distributions. Phys. Rev. A.

[41] Knapp M (2007). Complex interaction of hydrogen with the RuO_2_ (110) surface. J. Phys. Chem. C.

[42] Hohenberg P, Kohn W (1964). Inhomogeneous electron gas. Phys. Rev..

[43] Kohn W, Sham LJ (1965). Self-consistent equations including exchange and correlation effects. Phys. Rev..

[44] Perdew JP, Burke K, Ernzerhof M (1996). Generalized gradient approximation made simple. Phys. Rev. Lett..

[45] Blöchl PE (1994). Projector augmented-wave method. Phys. Rev. B.

[46] Kresse G, Joubert D (1999). From ultrasoft pseudopotentials to the projector augmented-wave method. Phys. Rev. B.

[47] Kresse G, Hafner J (1993). Ab initio molecular dynamics for liquid metals. Phys. Rev..

[48] Kresse G, Hafner J (1994). Ab initio molecular-dynamics simulation of the liquid-metal-amorphous-semiconductor transition in germanium. Phys. Rev. B.

[49] Kresse G, Furthmüller J (1996). Efficient iterative schemes for ab initio total-energy calculations using a plane-wave basis set. Phys. Rev. B.

[50] Zhu Q, Oganov AR, Glass CW, Stokes HT (2012). Constrained evolutionary algorithm for structure prediction of molecular crystals: methodology and applications. Acta Crystallogr..

[51] Lyakhov AO, Oganov AR, Stokes HT, Zhu Q (2013). New developments in evolutionary structure prediction algorithm USPEX. Comput. Phys. Commun..

[52] Monkhorst HJ, Pack JD (1976). Special points for Brillouin-zone integrations. Phys. Rev. B.

[53] Martínez, J. I., Hansen, H. A., Rossmeisl, J. & Nørskov, J. K. Formation energies of rutile metal dioxides using density functional theory. *Phys. Rev. B* **79** (2009).

[54] Becke AD (1992). Density-functional thermochemistry. II. The effect of the Perdew–Wang generalized-gradient correlation correction. J. Chem. Phys..

[55] *Handbook of chemistry and physics*. (CRC Press, 1996).

[56] Zhan C, Jiang D (2016). Understanding the pseudocapacitance of RuO_2_ from joint density functional theory. J. Phys. Condens. Matter.

